# Multigramme synthesis and asymmetric dihydroxylation of a 4-fluorobut-2*E*-enoate

**DOI:** 10.3762/bjoc.9.301

**Published:** 2013-11-26

**Authors:** James A B Laurenson, John A Parkinson, Jonathan M Percy, Giuseppe Rinaudo, Ricard Roig

**Affiliations:** 1 WestCHEM Department of Pure and Applied Chemistry, University of Strathclyde, Thomas Graham Building, 295 Cathedral Street, Glasgow G1 1XL, United Kingdom; 2 Carbosynth Ltd., 93 Innovation Drive, Milton Park, Abingdon, Oxfordshire OX14 4RZ, United Kingdom; 3 Department of Chemistry, University of Leicester, University Road, Leicester LE1 7RH, United Kingdom; 4 Lallemand Gb Ingredients, Dock Road, Felixstowe, Suffolk IP11 3QW, United Kingdom

**Keywords:** asymmetric, dihydroxylation, ee determination, fluorination, fluorosugars, organo-fluorine

## Abstract

Esters of crotonic acid were brominated on a multigramme scale using a free radical procedure. A phase transfer catalysed fluorination transformed these species to the 4-fluorobut-2*E*-enoates reproducibly and at scale (48–53%, ca. 300 mmol). Asymmetric dihydroxylation reactions were then used to transform the butenoate, ultimately into all four diastereoisomers of a versatile fluorinated C_4_ building block at high enantiomeric-enrichment. The (DHQ)_2_AQN and (DHQD)_2_AQN ligands described by Sharpless were the most effective. The development and optimisation of a new and facile method for the determination of ee is also described; ^19^F{^1^H} spectra recorded in *d*-chloroform/diisopropyl tartrate showed distinct baseline separated signals for different enantiomers.

## Introduction

Selective fluorination can be used to make subtle but decisive modifications of molecular properties. Sugar chemistry has proved particularly fertile ground for studies of this type; fluorine atoms can be used to replace hydroxy groups or hydrogen atoms, modifying the arrays of hydrogen bond donors and acceptors, and electron demand at the anomeric centre at minimal steric cost. Modifications of this type are sometimes accepted by sugar-processing enzymes such as the kinases and transferases involved in oligosaccharide assembly, or in antibiotic biosynthesis. Mechanistic insights, and new routes to hybrid natural products represent the rewards of this endeavour [1–10].

The synthesis of fluorinated analogues of sugars can be approached in two strategically different ways. The most common, and often most efficient approach, identifies a sugar precursor, isolates the locus for fluorination (usually an hydroxy group) by protecting all the other functional groups, and transforms it using a nucleophilic fluorinating agent [11].

**Scheme 1:**
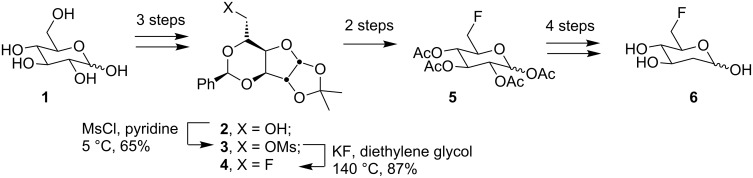
Key steps from the synthesis of 6-fluoro-D-olivose (**6**) from D-glucose (**1**).

The main advantages of this approach are that pre-existing stereogenic centres remain intact, while accurate inversion of configuration occurs at the locus of reaction. For one of the most common transformations, which delivers 6-deoxy-6-fluoro sugars, the locus of reaction is not even a stereogenic centre. The synthesis of 6-fluoro-D-olivose (**6**) in 23% overall yield from optically pure D-glucose (**1**) by O’Hagan and Nieschalk (Scheme 1) provides an impressive example of the approach [12].

Isolation of the C-6 hydroxy group in **2** set the stage for mesylation, and conversion of **3** to fluoride **4** with an extremely economical reagent. Acetal cleavage and peracetylation released glycoside **5** which was converted to **6** via known methods. The main disadvantages of the approach are the extensive use which must be made of protection/deprotection chemistry, and in some cases, the availability of the precursor sugar. Some less common sugars are expensive and available in limited quantities.

**Scheme 2:**
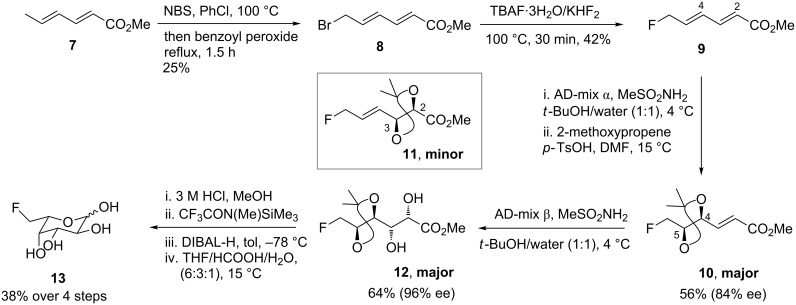
De novo asymmetric syntheses of 6-deoxy-6-fluorohexoses [13].

The alternative approach involves de novo stereodivergent synthesis, which elaborates small fluorinated building blocks using the reactions of modern catalytic asymmetric chemistry; this approach still has a very restricted repertoire. Few versatile building blocks are available, particularly in supra-millimol quantities, and other disadvantages include the need to carry an expensive fluorinated material through many steps, and requirements for chromatographic separations of diastereoisomers. The costs and benefits of the de novo approach were illustrated by our recent asymmetric, stereodivergent route to selected 6-deoxy-6-fluorohexoses in which we transformed a fluorinated hexadienoate **9** into the fluorosugars 6-deoxy-6-fluoro-L-idose, 6-fluoro-L-fucose (**13**, shown) and 6-deoxy-6-fluoro-D-galactose (Scheme 2) [13].

The main challenges we faced included the synthesis of **9** and its bromide precursor **8** in acceptable yield and purity, and the unexpectedly low regioselectivity of AD reactions of the fluorinated dienoate. Methyl sorbate (**7**) underwent AD across the C-4/C-5 alkenyl group exclusively, but the introduction of the fluorine atom at C-6 lowered the selectivity (**10**:**11**) to 5:1 with AD-mix-α and 4:1 with AD-mix-β.

Nevertheless, de novo stereodivergent approaches are conceptually important and pave the way to wider ranges of more unnatural species. We decided to solve the problem of low regioselectivity from the hexadienoate, and to discover a more stereodivergent repertoire, by attempting to develop asymmetric chemistry based on a smaller butenoate (C_4_) building block, **14**.

## Results and Discussion

**Scheme 3:**
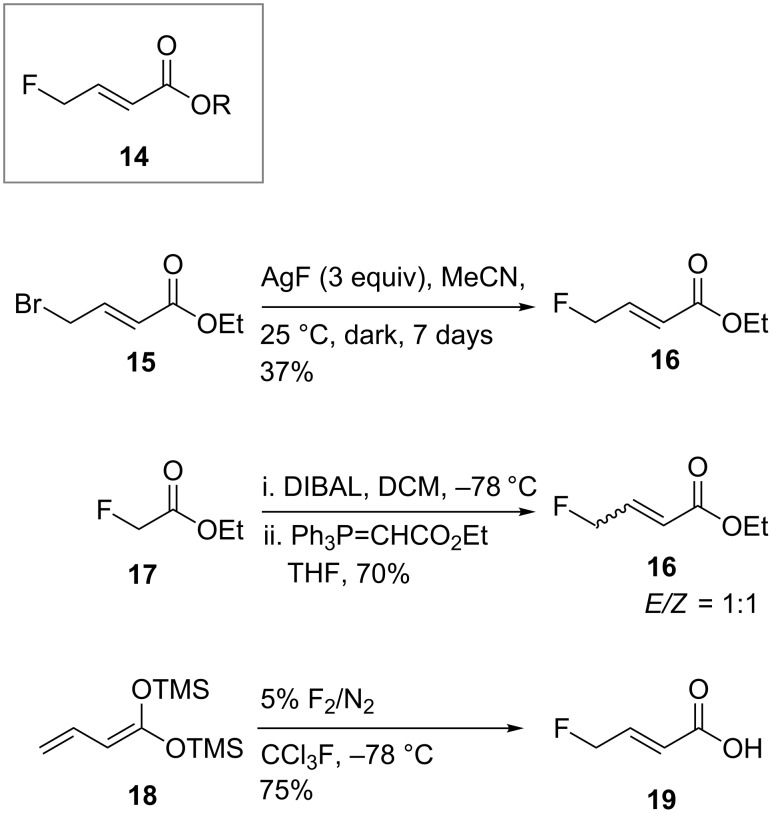
Fluorobutenoate building block **14**, and related species **16** and **19** from the literature [14–16].

Fluorides of type **14** are uncommon in the literature (Scheme 3); silver mediated fluorination of butenoyl bromide **15** is known [14] delivering **16** in moderate yield but via a slow and expensive reaction. Wittig reaction, following in situ reduction of ethyl fluoroacetate (**17**) has been reported [15], while Purrington [16] prepared **19** by direct fluorination of silylketene acetal **18** with elemental fluorine.

**Scheme 4:**
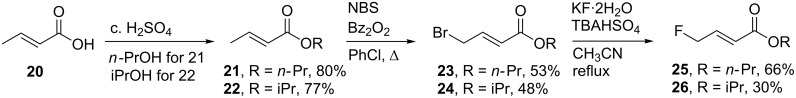
Fluorobutenoate building blocks **25** and **26** prepared from crotonic acid.

We decided to explore a halogen exchange approach from crotonic acid (**20**) which is commercially available cheaply, and in high diastereoisomeric purity (>98%). Diastereomeric purity is particularly important as the de novo syntheses must deliver the highest enantiomeric purity possible to be competitive with syntheses from enantiomerically pure natural products. *n*-Propyl and isopropyl esters **21** and **22** were prepared (0.5 mol scale) to moderate the volatility of intermediates, while retaining the option of distillation as a method of purification. Bromination was carried out using the method of Lester et al. [17], and while it was effective at small scales, larger scale (>150 mmol) reactions were violently exothermic. A modification of the reaction order reported earlier by Gershon and coworkers solved the problem [18]. Chlorobenzene was effective as the reaction solvent instead of carbon tetrachloride, allowing **23** and **24** to be isolated safely and reproducibly at scale (>300 mmol) in moderate yield (48–53%) after Kugelrohr distillation (Scheme 4).

Fluorination was attempted using a range of conditions. The solvent-free reaction developed within our laboratory using commercial TBAF and KHF_2_ was not sufficiently effective for this substrate [13,19]. The yield of the product was moderate (37%), but the purification of the product was extremely difficult due to the complex mixture of products.

**Figure 1:**
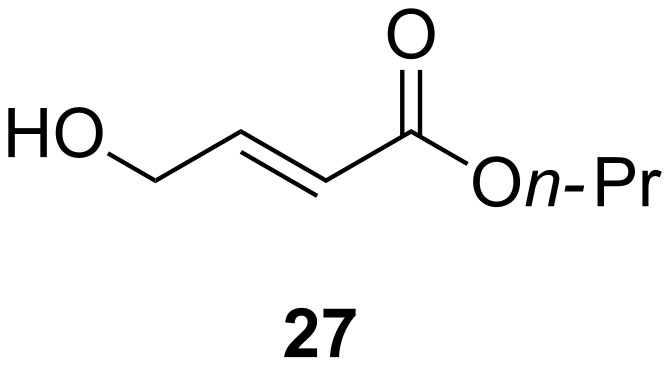
Side product **27** isolated from attempted fluorination.

Allyl alcohol **27** (Figure 1) and starting material **23** were present and difficult to separate. During the course of this project, TBAF·(*t-*BuOH)_4_ was reported to be more effective than other fluoride sources. Kim and co-workers [20] reported that the reagent was obtained as a non-hygroscopic crystalline white solid after refluxing commercial TBAF in a mixture of hexane and *t*-BuOH; importantly, they claimed that it can be considered as a truly anhydrous source of the TBAF reagent. We were completely unable to reproduce the reagent preparation reported in the literature; all the materials we were able to make were extremely hygroscopic indeed, and exposure of **23** or **24** to them resulted in complete decomposition to a very complex mixture of products. However, the phase transfer catalysed procedure described by Hou and co-workers [21] which used TBAHSO_4_ and KF·2H_2_O in refluxing acetonitrile successfully effected the fluorination to allyl fluorides **25** and **26** on both small and large scales (>150 mmol). Rapid Kugelrohr distillation under reduced pressure was attempted initially but the quality of the distilled material was unsatisfactory. Fractional distillation through a Vigreux column at reduced pressure yielded the desired fluorides in an acceptable level of purity (>95% by ^1^H NMR) and reproducibly on a large scale (up to ~200 mmol). These outcomes represent significant practical improvements on the published methods of preparation. The subsequent transformations were carried out on the *n*-propyl ester **25** for two reasons; firstly, the material can be made in much higher yield, and the *n*-propyl ester can be cleaved under milder conditions than the isopropyl ester in **26**.

Although the commercial AD-mixes (0.4 mol % osmium/1 mol % ligand) can transform most standard substrates smoothly, osmium tetroxide is an electrophilic reagent [22], and electron deficient olefins, such as unsaturated amides and esters, react relatively slowly [23].

**Figure 2:**
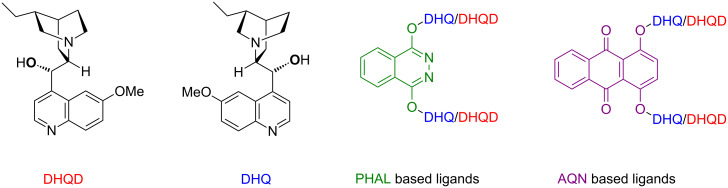
The ligand panel used in the asymmetric dihydroxylation studies. The bold oxygen shows the point of attachment; individual ligands are represented by combinations of components, for example (DHQD)_2_ PHAL, present in AD-mix β.

**Scheme 5:**
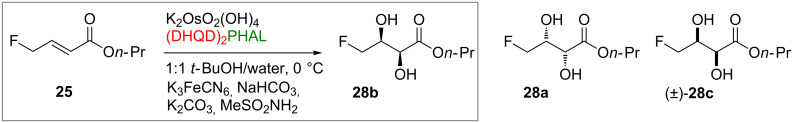
Typical AD procedure; see Table 1 for outcomes.

It was thought that the so-called “improved procedure” [24], which uses higher ligand/oxidant loadings (1 mol % osmium/5 mol % ligand) might be required to allow the reactions to proceed in acceptable yields and enantioselectivities [25]. Figure 2 shows the panel of ligands used for the asymmetric transformations. Scheme 5 shows the initial dihydroxylation carried out on **25**, and Table 1 summarises the method development.

**Table 1:** Relationship between conditions, ligand and dihydroxylation ee.

Conditions	Ligand type	DHQ/α-	DHQD/β-

Standard 0.4 mol % osmium, 1 mol % ligand	PHAL	66% ee	72% ee
2 mol % osmium, 2 mol % ligand	PHAL	80% ee	89% ee
Improved 1 mol % osmium, 5 mol % ligand	PHAL	83% ee	91% ee
1 mol % osmium, 10 mol % ligand	PHAL	82% ee	90% ee
1 mol % osmium, 5 mol % ligand	AQN	95% ee	97% ee

The asymmetric dihydroxylation conditions were subject to some optimization; the osmium and chiral ligand contents were varied in the first instance. While the commercial AD-mixes were used, we also carried out the dihydroxylations with 1 mol % osmium/5 mol % ligand, the so-called “improved procedure”, and with 1 mol % osmium/10 mol % ligand (results summarised in Table 1). Methyl sulfonamide which can accelerate hydrolysis and catalytic turnover was also added to the reaction mixtures [26].

Yields for the dihydroxylation chemistry were variable (44–80%); even though they are diols, these small molecules proved volatile. Reproducible yields (>55%) could be achieved if care was taken with solvent removal.

The “improved conditions” (1 mol % osmium, 5 mol % ligand) were found to give results comparable (within experimental error) to those obtained with the 2 mol % osmium/2 mol % ligand and 1 mol % osmium/10 mol % ligand conditions, suggesting the ee could not be indefinitely improved by increasing the ligand or osmium concentrations. Sharpless has reported that the (DHQ)_2_AQN and (DHQD)_2_AQN ligands based on the anthraquinone core, (Figure 2), are superior ligands for olefins bearing heteroatoms in the allylic position [27].

An asymmetric dihydroxylation reaction was performed using the improved Sharpless conditions with the newer AQN based ligands, producing excellent ee’s for both enantiomers of the diol, 95% for the enantiomer derived from AD-mix α, and 97% for the enantiomer from AD-mix β (Table 1). The corresponding isolated yields under these conditions were 54% and 56% respectively.

**Scheme 6:**
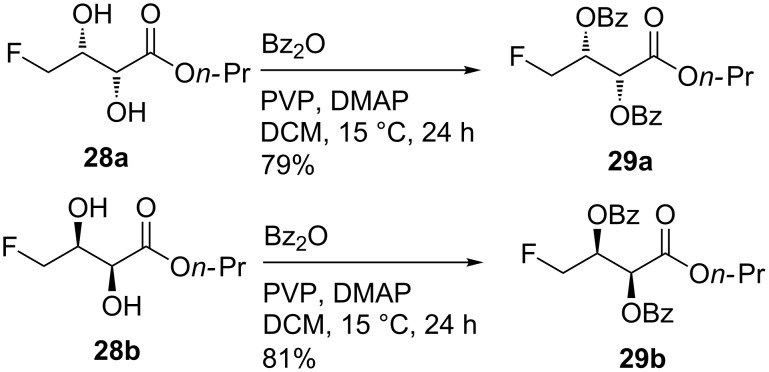
Conversion of enantiomerically-enriched diols to dibenzoates for HPLC analysis.

The ee's were measured after conversion of the diols to the dibenzoates **29** upon stirring overnight with benzoic anhydride, DMAP and polyvinylpyridine (PVP) at room temperature. The removal of the base by filtration was facile (Scheme 6). Genuine racemate **28c** was synthesised via the Upjohn oxidation (catalytic osmium tetroxide, NMO aqueous *t*-BuOH, 83%) of **25** to avoid ambiguity, and converted to the dibenzoate **29c** (not shown, 80%) as described above.

The dibenzoates were purified by flash chromatography then examined by chiral HPLC (Chiralcel OD, 2% iPrOH in hexane). The separation of the enantiomers **29a** and **29b** was excellent, with over 6 minutes separating the stereoisomers in the chromatograms. Due to the robust nature of the dibenzoylation chemistry and the excellent chromatograms produced, the derivatisation/chiral HPLC assay was used routinely.

However, direct measurement of the ee's of the fluorinated diols **28a** and **28b** could not be achieved by the HPLC method. The very low absorbance of light at 235 nm resulted in unreliable data; small peak areas were observed for the desired compound with comparatively large peak areas for the background and trace impurities (as judged by ^1^H and ^13^C NMR spectra). Attempts to use RI detection in the chiral HPLC were no more successful. A new analytical method was therefore sought which would allow the ee’s of the diols to be measured quickly and directly using ^19^F{^1^H} NMR, avoiding the introduction of additional synthetic steps.

**Figure 3:**
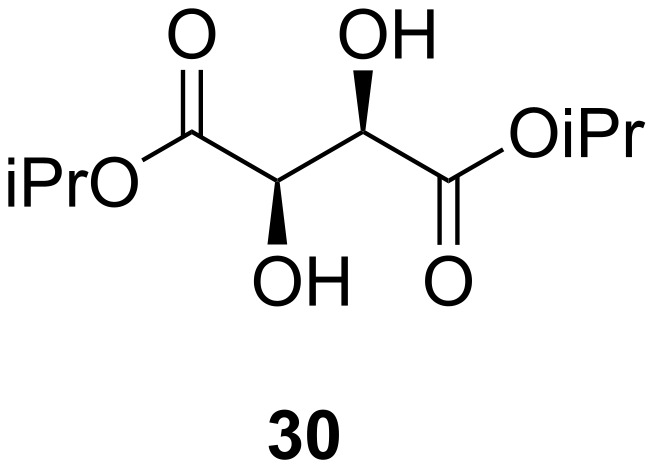
Diisopropyl L-tartrate (**30**) used as a chiral modifier for NMR determination of ee.

The determination of enantiomeric excesses using NMR is a well-established technique [28]; tactics include in situ derivatisation [29], may rely on very specific functionality [30] or may use expensive and/or structurally complex shift reagents [31]. The necessity of these reagents arises from the need to examine a single peak in a high level of detail despite the often cluttered nature of ^1^H (and ^13^C) NMR spectra, especially with large or complex structures. NMR determination of enantiomeric purity using chiral solvents though less well known has been described in the literature [32] and is particularly effective when heteroatomic NMR techniques are used [33]. For example, α-methylbenzylamine was used to resolve the components of the racemate of 2,2,2-trifluoro-1-phenylethanol in the ^19^F NMR spectrum (Δδ_F_ was 0.04 ppm) [34] and in another case, a chiral liquid crystalline medium was used to resolve racemic mixtures of fluoroalkanes very effectively [35]. When solubilised in a chiral environment like diisopropyl L-tartrate (**30**, Figure 3), the formation of diastereoisomeric solvation complexes results in magnetic non-equivalence and hence the appearance of separate signals for the complexes in the NMR experiment.

**Figure 4:**
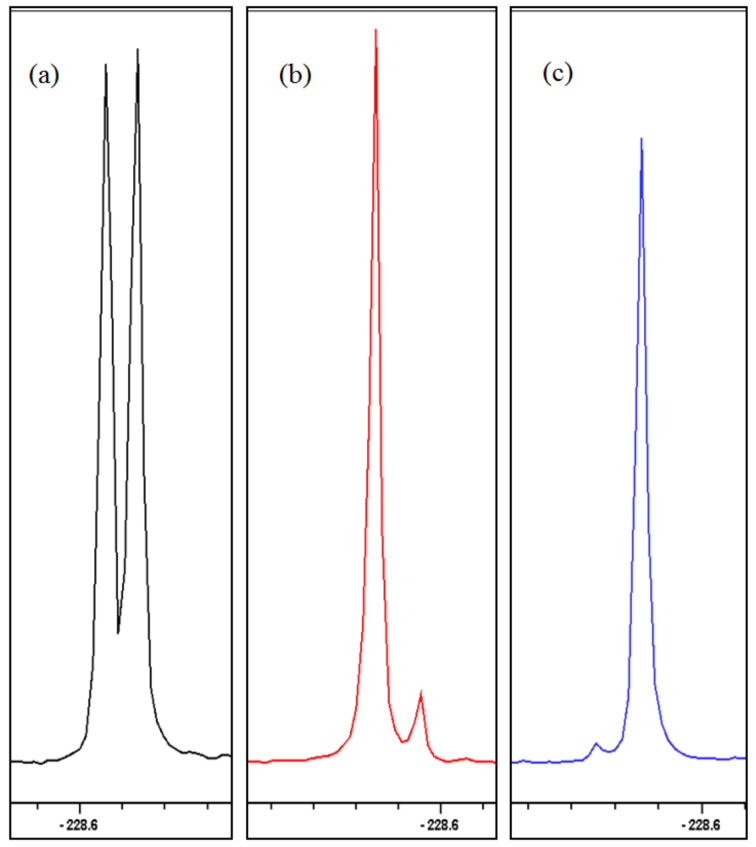
Partial ^19^F{^1^H} NMR spectra (376 MHz, L-(+)-DIPT/CDCl_3_, 300 K) spectra of (a) racemate **28c**, (b) diol **28b** and (c) **28a** under standard acquisition parameters revealing the partial enantiomer overlap.

Recording the ^19^F{^1^H} NMR spectra will take advantage of the high sensitivity of ^19^F NMR detection and optimise *S*/*N* through the removal of splittings to protons. The NMR experiment was performed by diluting the substrate in an NMR tube with a 1:1 w/w mixture of diisopropyl L-tartrate and CDCl_3_. Racemic diol **28c** analysed under these conditions by ^19^F{^1^H} NMR showed almost complete separation of the two enantiomers (Δδ_F_ = 0.02 ppm). However, more complete peak separation was required before reliable integrations could be made (Figure 4).

Alterations to the NMR acquisition parameters were made in an effort to improve the baseline resolution and separate the peaks fully.

**Figure 5:**
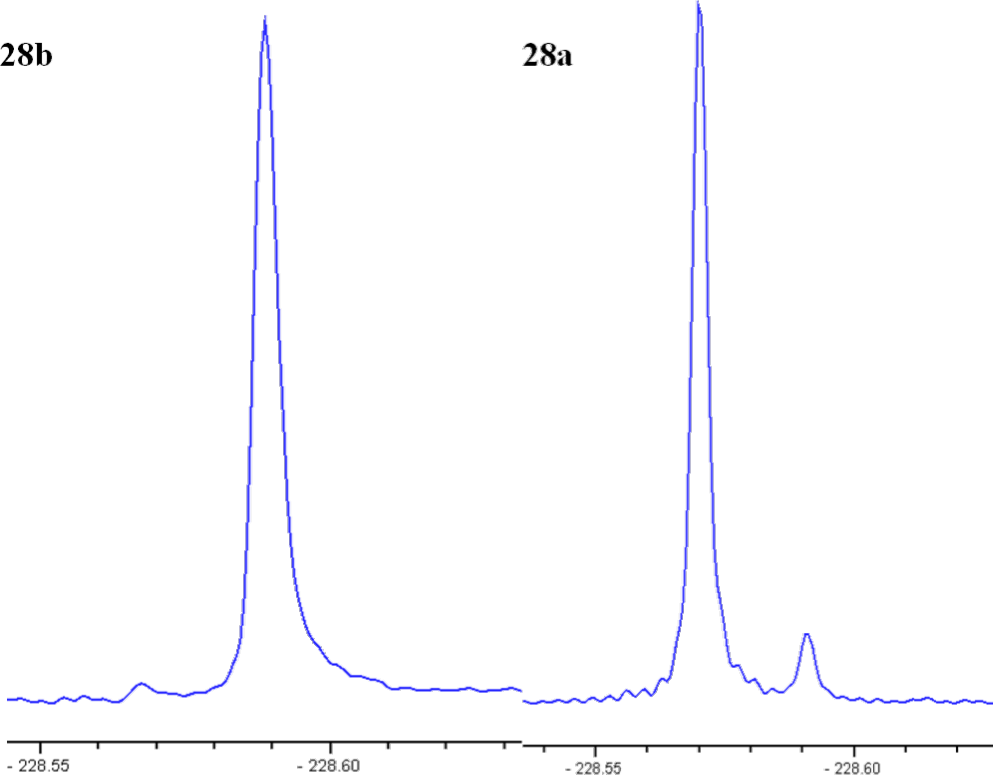
Partial ^19^F{^1^H} NMR (400 MHz, L-(+)-DIPT/CDCl_3_, 300 K) spectra of **28b** and **28a** using optimised conditions: SW 40; AQ = 0.8; O1P −230; d1 = 5; 32 or 64 scans.

Initial modifications caused a decrease in the quality of the spectra produced, with signal broadening and a reduction in the peak separation observed, caused by sample heating within the probe (decoupling produces heating of the sample) at the longer acquisition times. A set of experimental parameters that would allow a narrowing of the sweep width (SW), but maintain short acquisition (AQ) and relaxation times, and therefore minimise sample heating was devised; the optimised spectra are shown in Figure 5.

The results obtained from integration of the signals for each enantiomer matched the chiral HPLC analysis of the derivatised dibenzoates closely; for example the ee’s for **28b** and **28a**, from the 1 mol % osmium, 5 mol % PHAL conditions, were 82% and 91% by NMR respectively and 83% and 91% by HPLC for the corresponding dibenzoates **29b** and **29a**.

The ^19^F{^1^H} NMR method uses a cheap readily available chiral solvating agent, is rapid (2 minutes per sample) and simple to perform. Although the technique is sacrificial in the sample, the quantities of sample required (<2 mg) are negligible. We make no claims for the generality of the method, but for molecules of this type, it appears highly effective.

**Scheme 7:**
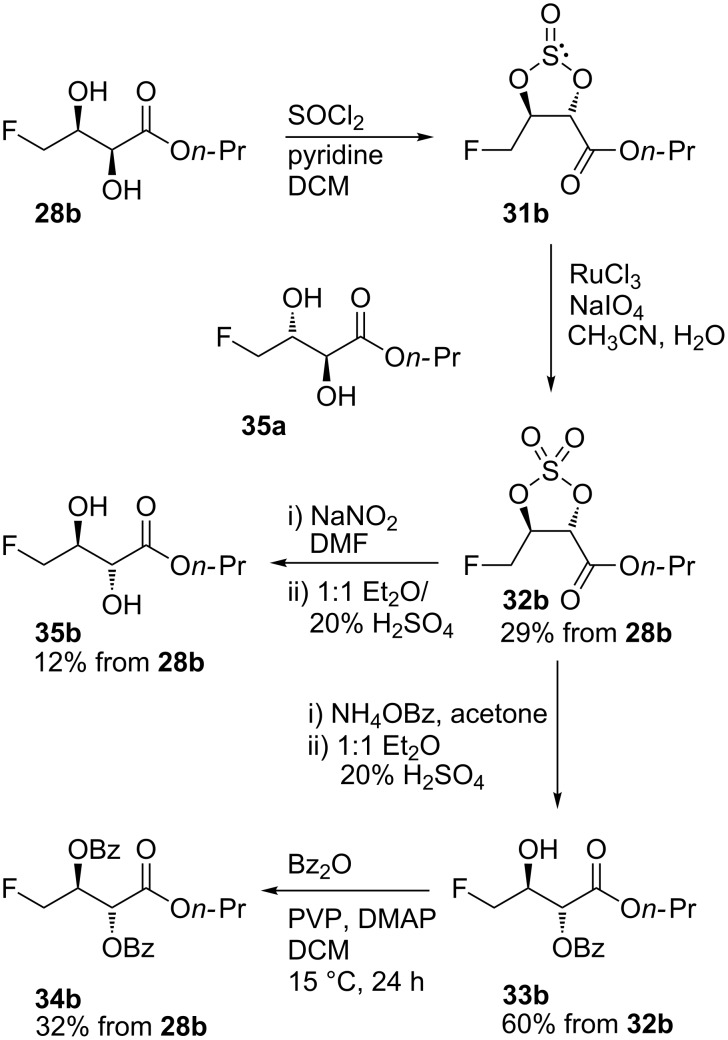
Applying cyclic sulfate methodology to gain access to *anti*-diastereoisomers (transformations were developed from racemic diol **28c**, but are shown for diol **28b** only).

To make our route stereodivergent, we sought access to the two *anti* diastereoisomers **35a** and **35b** via cyclic sulfate methodology (Scheme 7) [36–37].

Cyclic sulfate **32b** was prepared via literature procedures [36–37], monitoring the steps closely by ^19^F{^1^H} NMR spectroscopy which distinguishes all the species effectively. In **32b**, C-3 is primed for regioselective nucleophilic attack [38]. Crude cyclic sulfate **32b** was taken up in acetone, treated with solid ammonium benzoate and allowed to stir at room temperature overnight. Nucleophilic ring opening reactions were performed on the crude cyclic sulfate mixtures because avoiding column chromatography at this stage led to a vast improvement in the overall yields. After ring opening, sulfate ester cleavage was achieved by stirring the concentrated residue in acid (20% H_2_SO_4_) and ether, yielding the desired monobenzoate in moderate yield (60%) after purification. The regiochemistry of the ring opening was revealed in the HMBC spectrum of monobenzoate **33b**. The ^1^H NMR signal corresponding to the C-2 methine proton couples (^3^*J*_C-H_) to both carbonyl signals in the ^13^C spectrum. This indicates that both carbonyl groups are within 3 bonds of the hydrogen on C-2. However, the signal from the hydrogen on C-3 couples to the carbonyl carbon of the *n*-propyl ester only, confirming the expected regiochemistry for structure **33b**. Dibenzoate **34b** was synthesised (32% overall from **28b**) directly from the crude reaction mixture (Scheme 7) by treatment of the crude monobenzoate **33b** with benzoic anhydride in the presence of DMAP and PVP. The *syn-* and *anti-*dibenzoates have distinct signals in the ^19^F NMR spectra (δ_F_ −230.3 and −231.0 ppm respectively), allowing a very high level of confidence that the ring-opening of the *syn*-cyclic sulfates does not produce *syn*-dibenzoate, and that epimerisation is not competitive with ring-opening. This was further supported by chiral HPLC analyses of the dibenzoates, which also suggests that clean conversion occurs, without epimerisation. All four dibenzoates had distinct retention times in the chiral HPLC chromatograms.

For the inversion of the diol stereochemistry to be synthetically useful, a less basic synthetic equivalent for hydroxide was required. When Mitsunobu chemistry fails, O’Doherty and co-workers have achieved hydroxy group inversion by triflation and displacement using sodium nitrite [39]. Cyclic sulfate **32b** was exposed to sodium nitrite in DMF; the mixture was heated at reflux until completion of the reaction was confirmed by ^19^F NMR. Subsequent acid cleavage of the sulfate ester afforded the desired *anti-*diols in a disappointing yield (12% overall from **28b**) after purification. The low yield was attributed to the small scale of the reaction and difficulty of the work-up caused by the presence of DMF. Unfortunately, attempts to carry out the reaction in acetone led to complete decomposition of the substrate.

**Scheme 8:**
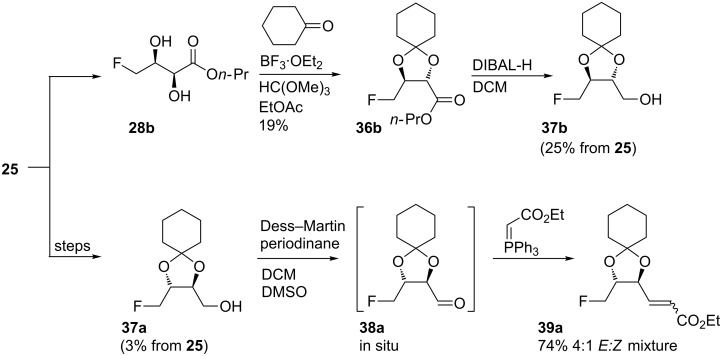
Protecting and chain extending the educts of asymmetric dihydroxylation.

A proof-of-concept extension sequence of the C_4_ building block was sought. Cyclohexylidene protection was chosen to add bulk and in aspiration to crystalline intermediates (Scheme 8).

After some initial failures, cyclohexylidene **36b** formed effectively in the presence of Lewis acid BF_3_·OEt_2_ in ethyl acetate [40]. Ester reduction with DIBAL-H afforded alcohol **37b**; delaying purification of the products until after the reduction step increased the overall yield from butenoate **25** to 25% over 3 steps and in excellent diastereoisomeric purity. In contrast, the preparation of **37a** with purifications at each stage delivered **37a** in 3% overall yield. A one-pot oxidation/Wittig procedure was implemented from **37a**; treatment with the Dess–Martin periodinane [41] in the presence of the stabilised ylide afforded a 4:1 *E:Z* mixture of the product alkene **39a** in good (74%) yield. A second purification by column chromatography isolated the *E*-alkene diastereoisomer of **39a** in 37% yield together with a mixed fraction of the *E-* and *Z-*alkenes. The *E-*isomer was identified by the alkene vicinal coupling values in the ^1^H NMR spectrum, and *E:Z* ratios were measured by integration of the distinct signals in the ^19^F{^1^H} NMR spectra. Analysis of the pure *E-*alkene using the chiral ^19^F{^1^H} NMR method revealed that the ee was unchanged from the diol **28a**, confirming epimerisation was not occurring during the subsequent reactions (aldehyde **38a** was of particular concern).

The synthesis of alkenes **39** is particularly significant, as at this stage the crotonic acid route overlaps with the published syntheses of 6-deoxy-6-fluorohexoses from methyl sorbate [13].

The main benefits of the crotonic acid route are the absence of regioisomers as the double bond is installed after the asymmetric oxidation and the potential to deliver all of the 6-deoxy-6-fluorohexose isomers, as the cyclic sulfate chemistry can generate the previously inaccessible *anti-*diol relationships, either at C2–C3, C4–C5 or both.

## Conclusion

A practical route which affords 4-fluorobut-2*E*-enoates reproducibly and at scale (48–53%, ca. 300 mmol) has been developed, improving significantly on published methods. Catalytic asymmetric dihydroxylation can be carried out in moderate to good yields and in excellent ee using the AQN ligands. Chiral HPLC was used for ee determination of the dibenzoate derivatives, but a chiral ^19^F{^1^H} NMR method was developed to determine the enantiomeric purities of the non-chromophoric *syn*-diol products. Educt elaboration was achieved via cyclic sulfate methodology, leading to the stereocomplementary *anti*-diols, and via acetal protection, ester reduction and one-pot oxidation/Wittig reaction, re-connecting this study to the published route to 6-deoxy-6-fluorohexoses.

## Experimental

A full range of experimental procedures and characterisation data is presented in Supporting Information File 1.

## Supporting Information

File 1 Experimental procedures and characterization data.
