# Supplementary material for: Multigramme synthesis and asymmetric dihydroxylation of a 4-fluorobut-2E-enoate
Source: Beilstein J Org Chem. 2013 Nov 26;9:2660–8. doi: 10.3762/bjoc.9.301 (PMC3869297; doi:10.3762/bjoc.9.301)
Supplement: File 1 — Experimental procedures and characterization data. [file Beilstein_J_Org_Chem-09-2660-s001.pdf]

Supporting Information

for

Multigramme synthesis and asymmetric dihydroxylation of a 4-fluorobut-2*E*-enoate

James A. B. Laurenson[1,2], John A. Parkinson[1], Jonathan M. Percy*[1], Giuseppe Rinaudo[1] and Ricard Roig[3,4]

Address: [1]WestCHEM Department of Pure and Applied Chemistry, University of Strathclyde, Thomas Graham Building, 295 Cathedral Street, Glasgow G1 1XL, United Kingdom, [2]Carbosynth Ltd., 93 Innovation Drive, Milton Park, Abingdon, Oxfordshire OX14 4RZ, United Kingdom, [3]Department of Chemistry, University of Leicester, University Road, Leicester LE1 7RH, United Kingdom and [4]Lallemand Gb Ingredients, Dock Road, Felixstowe, Suffolk IP11 3QW, United Kingdom

Email: Jonathan M. Percy - jonathan.percy@strath.ac.uk

* Corresponding author

Experimental procedures and characterization data

General synthetic procedures

NMR spectra were recorded on a Bruker AV400 spectrometer (^{1}H 400.03 MHz; ^{13}C 100.59 MHz; ^{19}F 376.40 MHz) or a Bruker DPX400 spectrometer (^{1}H 400.13 MHz; ^{13}C 100.59 MHz; ^{19}F 376.50 MHz). NMR spectra were recorded using deuterated solvent as the lock and residual solvent as the internal reference. The multiplicities of the spectroscopic data are presented in the following manner: app. = apparent, s = singlet, d = doublet, t = triplet, dd = double doublet, td = triplet doublets, br = broad, m = multiplet. The appearance of complex signals is indicated by app. Homocouplings (H–H) are given in Hertz and specified by *J*; the nuclei involved in heteronuclear couplings are defined with the observed nucleus given first. Unless stated otherwise, all refer to ^{3}J couplings. Low resolution ESI analyses were performed on a Finnigan LC Q Duo mass spectrometer. GC–MS analyses were performed on a Finnigan Polaris Q spectrometer carried on a 30 m × 0.25 µm PE-5 column running a 40–320 °C ramp over 25 minutes (unless otherwise stated). High resolution mass spectrometry measurements were carried out at the EPSRC National Mass Spectrometry Service Centre in Swansea using peak matching to suitable reference peaks, depending on the technique used. Thin Layer Chromatography (TLC) was performed on pre-coated aluminium backed silica gel plates (Merck, silica gel 60 F_{254}). The compounds were visualized using UV light (λ = 254 and 386 nm), potassium permanganate or *p*-anisaldehyde stains. Flash chromatography was performed using silica gel (33–70 µm) and a Buchi Sepacore system. Infra-red (IR) spectra were obtained on a Perkin Elmer Spectrum One FTIR spectrometer. Melting points were obtained using a Reichert hot stage melting point apparatus. Optical rotations were performed on a Perkin Elmer Polarimeter 341 using an optical cell (path length 10 cm) maintained at 298 K with a continuous flow

water bath. Solvents were dried (when required) using a PureSolv apparatus (Innovative Technologies Inc.). All other chemicals and solvents were used as received from suppliers without any further purification.

CAUTION All the materials made in this study were treated **with the utmost caution**; all skin contact and inhalation were avoided with the greatest care. The presence of the fluorine atom at the chain end may result in the release of fluoroacetic acid, **a fatal poison**, upon metabolism. Esters **23** and **24** were viewed as particularly hazardous because of their volatility; diaphragm pumps used on rotary evaporators were protected by Dreschel bottles filled with bleach, and rotary pump exhaust was vented carefully into a fume cupboard. Rotary pump oil was changed and disposed of upon completion of the work.

Determination of ee by $^{19}F\{^{1}H\}$ NMR procedure

The sample to be analysed was dissolved (~2–3 mg/mL) in a mixture of $CDCl_3$ and di*iso*propyl L-tartrate (1:1 w/w) and an aliquot (0.75 mL) transferred to an NMR tube. The spectrometer acquisition parameters were set to:

SW = 40 (sweep width); AQ = 0.8 (acquisition time); O1P = −230 (spectral window centre); d1 = 5 seconds (relaxation delay). Upon completion of the NMR experiment the signals for each enantiomer in the resulting spectrum were integrated to determine the ee of the sample.

Preparation of *n*-propyl but-2-*E*-enoate (21)

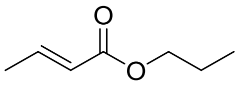

A solution of crotonic acid (**20**, 581 mmol, 50 g) in *n*-propanol (225 mL) containing concentrated sulfuric acid (6.9 mL) was heated at reflux overnight. The reaction

mixture was then diluted with diethyl ether (100 mL) and washed with aqueous $NaHCO_3$ (ca. 3 × 75 mL of a saturated aqueous solution) until no more gas was evolved. The combined aqueous washings were back extracted with diethyl ether (2 × 100 mL) and the combined organic extracts and original layer were dried ($MgSO_4$) and concentrated in vacuo to afford ester **21** as a colourless oil which was used in the next step without purification (59.7 g, ca. 80%). The following data were obtained from a small purified sample (flash chromatography, 10% diethyl ether in hexane): R_f (12.5% diethyl ether in pet. ether 40–60 °C) 0.66; δ_H (400 MHz, $CDCl_3$) 6.97 (dq, J = 15.7, 7.0, 1H), 5.84 (dq, J = 15.7, 1.8, 1H), 4.1 (t, J = 7.0, 2H), 1.87 (dd, J = 7.0, 1.8, 3H), 1.72-1.63 (m, 2H), 0.95 (t, J = 7.0, 3H); δ_C (100 MHz, $CDCl_3$) 166.6, 144.3, 122.7, 65.7, 22.0, 17.8, 10.3; *m/z* (EI) 129 (15%), 87 (35), 69 (100); HRMS (EI^+, $[M]^+$) calcd. for $C_7H_{12}O_2$ 128.0831, found 128.0832. The ester was reported previously [S1] but at a lower level of characterisation.

Preparation of isopropyl but-2-*E*-enoate (22)

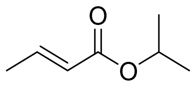

Was prepared from crotonic acid **20** (872 mmol, 75 g), isopropanol (365 mL) and concentrated sulfuric acid (8.8 mL) according to the previous procedure, work-up and isolation, to afford ester **22** (86.5 g ca. 77%) as a colourless oil which was used in the next step without purification. The following data were obtained from a small purified sample (flash chromatography, 10% diethyl ether in hexane): R_f (20% diethyl ether in hexane) 0.59; δ_H (400 MHz, $CDCl_3$) 7.00-6.89 (m, 1H), 5.82 (d, J = 15.7, 1H), 5.10-5.02 (m incl. app. septet, J = 6.3, 1H), 1.86 (d, J = 6.3, 3H), 1.25 (d, J = 6.3, 6H); δ_C(100 MHz, $CDCl_3$) 166.1, 144.0, 123.3, 67.3, 21.9, 17.8; ν_{max}(film)/cm^{-1} 3539, 3422, 2981, 2878, 1720, 1660, 1446, 1375, 1293, 1191, 1110, 1002, 834, 690;

HRMS (EI^+, $[M]^+$) calcd. for $C_7H_{12}O_2$ 128.0832, found 128.0830. The data were in agreement with those reported by Jonczyk et al. [S2].

Preparation of *n*-propyl 4-bromobut-2-*E*-enoate (23)

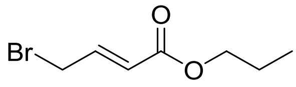

A solution of *N*-bromosuccinimide (41.7 g, 234 mmol), benzoyl peroxide (283 mg, 1.17 mmol) and ester **21** (30 g, 234 mmol) in chlorobenzene (300 mL) was warmed slowly to 85 °C over ca. 45 minutes. The reaction mixture was then allowed to stir at this temperature for 2.5 days. The cooled reaction mixture was then filtered and the precipitate washed with diethyl ether (200 mL). The combined filtrate and washings were then washed with NaOH (4 × 200 mL of a 5% aqueous solution) until the washings were almost colourless, then with brine (200 mL), dried ($MgSO_4$) and concentrated in vacuo to afford an orange oil. Kugelrohr distillation (80 °C/0.38 mmHg) afforded the bromide **23** as a pale yellow oil (25.97 g, ca. 53% yield) which was used in the next step without further purification (estimated to be ~90% pure by ^{1}H NMR). The following data were obtained from a small purified sample (flash chromatography, 10% diethyl ether in hexane): R_f (20% diethyl ether in hexane) 0.59; δ_H (400 MHz, $CDCl_3$) 7.00 (dt, *J* = 15.4, *J* = 7.3, 1H), 6.04 (dt, *J* = 15.4, *J* = 1.3, 1H), 4.11 (t, *J* = 6.8, 2H), 4.01 (dd, *J* = 7.3, *J* = 1.3, 2H), 1.75-1.64 (m, 2H), 0.96 (t, *J* = 7.3, 3H); δ_C (100 MHz, $CDCl_3$) 165.4, 141.5, 124.6, 66.2, 29.1, 21.9, 10.3; ν_{max}(film)/cm^{-1} 2969, 2879, 1722 (C=O), 1655 (C=C); *m/z* (EI^+) 207 (100%), 147 (50), 68 (30); HRMS (EI^+, $[M]^+$) calcd for $C_7H_{11}BrO_2$ 205.9934, found 205.9931; Anal. Calcd. for $C_7H_{11}BrO_2$: C, 40.60; H, 5.35; Br, 38.59. Found: C, 40.51; H, 5.12; Br, 38.41.

Preparation of isopropyl 4-bromobut-2-*E*-enoate (24)

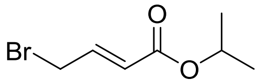

Was prepared from a solution of *N*-bromosuccinimide (60.7 g, 341 mmol), benzoyl peroxide (362 mg, 1.5 mmol) and ester **22** (39.7 g, 310 mmol) in chlorobenzene (200 mL) and according to the previous procedure, work-up and isolation the bromide **24** was obtained (33.8 g, ca 48%). The following data were obtained from a small purified sample (Flash chromatography, 0–25% diethyl ether in hexane): R_f (10% diethyl ether in hexane) 0.28; δ_H (400 MHz, $CDCl_3$) 7.02-6.94 (m, 1H), 6.06-6.01 (m incl. app. dt, J = 15.2, J = 1.1, 1H), 5.12-5.04 (sept., J = 6.3, 1H), 4.01 (dd, J = 7.3, 1.1, 2H), 1.27 (d, J = 6.3, 6H); δ_C (100 MHz, $CDCl_3$) 165.0, 141.3, 125.2, 68.2, 29.2, 21.8; ν_{max}(film)/cm^{-1} 3419, 2982, 2939, 1718, 1654, 1468, 1313, 1280, 1197, 1108, 976, 909, 823, 722, 584; *m/z* (EI^+) 209 (85), 207 (100), 149 (24), 109 (7), 81 (14), 68 (21); HRMS (EI^+, $[M]^+$) calcd. for $C_7H_{11}BrO_2$ 205.9937, found 205.9936.

Preparation of *n*-propyl 4-fluorobut-2-*E*-enoate (25)

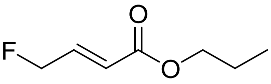

Bromoester **23** (22.05 g, 106 mmol) was added to a solution of $KF{\cdot}2H_2O$ (39.9 g, 424 mmol) and Bu_4NHSO_4 (43.2 g, 127 mmol) in acetonitrile (500 mL) and the mixture was heated at reflux overnight. The mixture was then diluted with water (400 mL) and extracted with diethyl ether (1 × 400 mL and 2 × 150 mL). The combined organic extracts were washed with brine (300 mL), dried ($MgSO_4$) and concentrated in vacuo, then fractionally distilled under reduced pressure (55–70 °C/0.08 mmHg) to afford fluoride **25** (10.2 g ca. 66%) as a pale yellow oil. The material (estimated to be ~90% pure by ^1H NMR) was used in subsequent reactions. The following data were

obtained from a small purified sample (Flash chromatography, 10% Et_2O in hexane): R_f (20% diethyl ether in hexane) 0.55; δ_H (400 MHz, $CDCl_3$) 6.96 (ddt, $^3J_{H\text{-}F}$ = 22.7, J = 15.9, 3.8, 1H), 6.12 (dq, J = 15.9, $^4J_{H\text{-}F}$ = 2.0, 1H), 5.06 (ddd, $^2J_{H\text{-}F}$ = 46.2, J = 3.8, 2.0, 2H), 4.12 (t, J = 6.7, 2H), 1.75-1.65 (m, 2H), 0.96 (t, J = 7.3, 3H); δ_C (100 MHz, $CDCl_3$) 165.8, 141.3 (d, $^2J_{C\text{-}F}$ 16.0), 121.4 (d, $^3J_{C\text{-}F}$ 11.4), 81.1 (d, $^1J_{C\text{-}F}$ 171.3), 66.2, 21.9, 10.3; δ_F (376 MHz, $CDCl_3$) -223.3 (tdd, $^2J_{F\text{-}H}$ 46.2, $^3J_{F\text{-}H}$ 22.7, $^4J_{F\text{-}H}$ 2.0); ν_{max}(film)/cm^{-1} 2971, 2882, 1722 (C=O), 1669 (C=C); m/z (EI^+) 163 (8%), 147 (80), 87 (100), 59 (19); HRMS (EI^+, $[M]^+$) calcd. for $C_7H_{11}FO_2$ 146.0738, found 146.0736, (EI^+, $[M + H]^+$) calcd. for $C_7H_{12}FO_2$ 147.0816, found 147.0815.

Preparation of isopropyl 4-fluorobut-2-*E*-enoate (26)

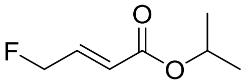

Was prepared from bromoester **24** (34.5 g, 167 mmol), KF·$2H_2O$ (62.1 g, 668 mmol) and Bu_4NHSO_4 (67.22 g, 200 mmol) in acetonitrile (650 mL) according to the previous procedure, work-up and isolation. Fractional distillation under reduced pressure (55–70 °C/0.08 mmHg) afforded ester **26** as a pale yellow oil (7.34 g, 30%, estimated to be ~95% pure by ^1H NMR) was used in subsequent reactions without any further purification. The following data were obtained from a small purified sample (flash chromatography, 10% diethyl ether in hexane): R_f (10% diethyl ether in hexane) 0.22; δ_H (400 MHz, $CDCl_3$) 6.99-6.86 (m, 1H), 6.11-6.04 (m incl. app. d, J = 15.7, 1H), 5.11-4.96 (m, 3H), 1.26 (d, J = 6.7, 6H); δ_C (100 MHz, $CDCl_3$) 165.2, 141.0 (d, $^2J_{C\text{-}F}$ = 17.6), 122.0 (d, $^3J_{C\text{-}F}$ = 11.4), 81.1 (d, $^1J_{C\text{-}F}$ = 172.7), 68.1, 21.8; δ_F (376 MHz, $CDCl_3$) -230.1 (tdd, $^2J_{F\text{-}H}$ = 46.5, $^3J_{F\text{-}H}$ = 22.4, $^4J_{F\text{-}H}$ = 1.7); ν_{max}(film)/cm^{-1} 3430, 2983, 2878, 1721, 1667, 1469, 1376, 1305, 1279, 1182, 1108, 999, 912, 836, 680, 587; m/z (EI^+) 233 (10%), 147 (100), 87 (80), 59 (10); HRMS (EI^+, $[M]^+$) calcd. for

$C_7H_{11}FO_2$ 146.0738, found 146.0738, (EI^+, $[M + H]^+$) calcd. for $C_7H_{12}FO_2$ 147.0816, found 147.0815.

Preparation of *n*-propyl (2*S,3*S**)-dihydroxy-4-fluorobutanoate ((±)-28c); Upjohn dihydroxylation of 25**

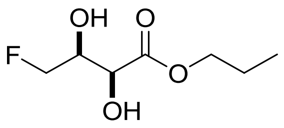

A solution of NMO (1.4 g, 11.4 mmol) in water (1 mL) was added to a mixture of *t*-BuOH and acetone (10 mL of a 1:1 mixture) at 0 °C. Ester **25** (836 mg, 5.7 mmol) was then added in one portion, the mixture allowed to stir for 15 minutes and then OsO_4 (1.4 mL of a 2.5 wt % solution in *t*-BuOH) was added dropwise by syringe over 20 minutes. The mixture was allowed to continue stirring and warm slowly to room temperature overnight. The reaction was quenched by the addition of sodium sulfite (8 g) and diluted with water (30 mL). The aqueous solution was then extracted with EtOAc (2 × 30 mL) and the combined organic extracts were dried ($MgSO_4$) and concentrated in vacuo to afford diol **28c** as an orange gum that was used without purification (855 mg, ca. 83%). The following data were obtained from a small purified sample (flash chromatography, 1% methanol in 1:1 ethyl acetate/hexane): R_f (50% ethyl acetate in hexane) 0.47; δ_H (400 MHz, $CDCl_3$) 4.50 (ddd, $^2J_{H\text{-}F}$ = 46.6, J = 9.4, 6.1, 2H), 4.27 (br s, 1H), 4.25-4.13 (m, 3H), 3.33 (br s, 1H), 2.85 (br s, 1H), 1.70 (sextet, J = 7.2, 2H), 0.95 (t, J = 7.2, 3H); δ_C (100 MHz, $CDCl_3$) 172.8, 83.0 (d, $^1J_{C\text{-}F}$ = 170.6), 70.5 (d, $^2J_{C\text{-}F}$ = 21.0), 70.0 (d, $^3J_{C\text{-}F}$ = 5.1), 68.0, 21.8, 10.2; δ_F (376 MHz, $CDCl_3$) -225.5 (td, $^2J_{F\text{-}H}$ = 46.9, $^3J_{F\text{-}H}$ = 13.4); ν_{max}(film)/cm^{-1} 3454, 2982, 2865, 1756, 1451, 1375, 1287, 1220, 1105, 927; HRMS (ES^+, $[M + H]^+$) calcd. for $C_7H_{14}FO_4$ 181.0871, found 181.0871; Anal. Calcd.: C, 46.66; H, 7.27; Found C, 46.34; H, 7.30.

Preparation of *n*-propyl (2*S*,3*S*)-dihydroxy-4-fluorobutanoate (28b); Sharpless asymmetric dihydroxylations of 25

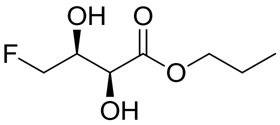

Sharpless AD with 2 mol % osmium/2 mol % ligand

A solution of $K_3Fe(CN)_6$ (2 g, 6.09 mmol), K_2CO_3 (840 mg, 6.09 mmol), $MeSO_2NH_2$ (195 mg, 2.03 mmol), $(DHQD)_2PHAL$, (2 mol %), $K_2OsO_4\cdot 2H_2O$ (2 mol %) in a mixture of *t*-BuOH and water (14 mL, 1:1 v/v) was cooled to 0 °C in a jacketed flask (connected to circulating chiller) and allowed to stir for ca. 30 min until a homogenous solution with a deep orange colour was observed. Fluoroester **25** (300 mg, 2.03 mmol) was then added in one portion and the mixture was allowed to stir at 0 °C for 15 hours (reaction completion was confirmed by micro work-up of an aliquot and analysis by ^{19}F NMR). The reaction was quenched by the addition of solid sodium sulfite (2 g) and allowed to warm to room temperature. A colour change from yellow to grey was observed. Water (30 mL) was then added and the suspension was extracted with ethyl acetate (3 × 30 mL). The combined organic extracts were then washed with brine (30 mL), KOH (30 mL of a 1 M solution), dried ($MgSO_4$) and concentrated in vacuo. Purification by flash chromatography (Buchi Sepacore, 1% methanol in 1:1 ethyl acetate/hexane) afforded diol **28b** as a colourless oil (163 mg, 44%). Data were identical to those described previously for **28c** apart from; $[\alpha]_D$ -13.2 (*c* 1.0, $CDCl_3$), 89% ee by $^{19}F\{^1H\}$ NMR, L-(+)-DIPT/$CDCl_3$.

General procedure for AD-mix β standard conditions

Crude diol **28b** was prepared from a solution of fluoroester **25** (1.04 g, 7.1 mmol), AD-mix β (9.94 g) and methyl sulfonamide (675 mg, 7.1 mmol) in 1:1 *t*-BuOH/water (70 mL) according to the procedure and work-up described previously. Purification by flash chromatography (Buchi Sepacore, 50% diethyl ether in hexane) afforded diol **28b** as a colourless oil (700 mg, 54%, 72% ee by $^{19}F\{^1H\}$ NMR, L-(+)-DIPT/$CDCl_3$).

1 mol % osmium/10 mol % ligand

Diol **28b** was prepared from a solution of ester **25** (73 mg, 0.5 mmol), AD-mix β (700 mg), methyl sulfonamide (48 mg, 0.5 mmol), $(DHQD)_2$PHAL (9 mol %), $K_2OsO_2(OH)_4$ (0.6 mol %) and $NaHCO_3$ (1.5 mmol) in *t*-BuOH/water (5 mL, 1:1 v/v) according to the procedure and work-up described previously to afford diol **28b** (71 mg, ca. 79%, 90% ee by $^{19}F\{^1H\}$ NMR, L-(+)-DIPT/$CDCl_3$).

General procedure for modified AD-mix β conditions

1 mol % osmium/5 mol % ligand

Crude diol **28b** was prepared from a solution of fluoroester **25** (2.19 g, 15 mmol), AD-mix β (21 g), methyl sulfonamide (1425 mg, 15 mmol), $(DHQD)_2$PHAL (4 mol %), $K_2OsO_2(OH)_4$ (0.6 mol %) and $NaHCO_3$ (45 mmol) in 1:1 *t*-BuOH/water (150 mL) according to the procedure and work-up described previously to afford diol **28b** (1.87 g, 78%, 91% ee by $^{19}F\{^1H\}$ NMR, L-(+)-DIPT/$CDCl_3$).

1 mol % osmium/5 mol % (DHQD)$_2$AQN ligand

Crude diol **28b** was prepared from a solution of fluoroester **25** (3 g, 20.3 mmol), $K_3Fe(CN)_6$ (20 g, 60.9 mmol), K_2CO_3 (8.4 g, 60.9 mmol), $MeSO_2NH_2$ (1.95 g, 20.3 mmol), (DHQD)$_2$AQN, (5 mol %, 870 mg), $K_2OsO_4{\cdot}2H_2O$ (2 mol %, 74 mg) in a mixture of *t*-BuOH and water (140 mL, 1:1 v/v) according to the procedure and work-up described previously to afford diol **28b** (2.63 g, 72%, 97% ee by $^{19}F\{^1H\}$ NMR, L-(+)-DIPT/$CDCl_3$).

Preparation of *n*-propyl (2*R*,3*R*)-dihydroxy-4-fluorobutanoate (28a)

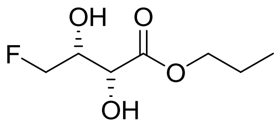

General procedure for AD-mix α standard conditions

Crude diol **28a** was prepared from a solution of fluoroester **25** (1.04 g, 7.1 mmol), AD-mix α (9.94 g) and methyl sulfonamide (675 mg, 7.1 mmol) in 1:1 *t*-BuOH/water (70 mL) according to the procedure and work-up described previously. Purification by flash chromatography (Buchi Sepacore, 50% diethyl ether in hexane) afforded the desired product **28a** as a colourless oil (712 mg, 56%, 66% ee by $^{19}F\{^1H\}$ NMR, L-(+)-DIPT/$CDCl_3$).

Sharpless AD with 2 mol % osmium/2 mol % ligand

Crude diol **28a** was prepared from a solution of fluoroester **25** (302 mg, 2.07 mmol), $K_3Fe(CN)_6$ (2.04 g, 6.21 mmol), K_2CO_3 (858 mg, 6.21 mmol), $MeSO_2NH_2$ (198 mg, 2.07 mmol), (DHQ)$_2$PHAL, (2 mol %), $K_2OsO_4.2H_2O$ (2 mol %) in *t*-BuOH/water (14 mL, 1:1 v/v) according to the procedure and work-up described previously. Purification by flash chromatography (Buchi Sepacore, 2% methanol in 1:1 ethyl acetate/hexane) afforded diol **28a** as a colourless oil (163 mg, 45%). Data were

identical to those described previously for **28c** apart from; $[\alpha]_D$ +12.8 (*c* 1.0, $CDCl_3$), 80% ee by $^{19}F\{^1H\}$ NMR, L-(+)-DIPT/$CDCl_3$.

General procedure for modified AD-mix α conditions:

1 mol % osmium/10 mol % ligand

Crude diol **28a** was prepared from a solution of fluoroester **25** (73 mg, 0.5 mmol), AD-mix α (700 mg), methyl sulfonamide (48 mg, 0.5 mmol), $(DHQ)_2PHAL$ (9 mol %), $K_2OsO_2(OH)_4$ (0.6 mol %) and $NaHCO_3$ (1.5 mmol) in *t*-BuOH/water (5 mL, 1:1 v/v) according to the procedure and work-up described previously to afford diol **28a** (54 mg, ca. 60%, 82% ee by $^{19}F\{^1H\}$ NMR, L-(+)-DIPT/$CDCl_3$).

General procedure for modified AD-mix α conditions:

1 mol % osmium/5 mol % ligand

Crude diol **28a** was prepared from a solution of fluoroester **25** (1.6g, 10.8 mmol), AD-mix α (15.1 g), methyl sulfonamide (1027 mg, 10.8 mmol), $(DHQ)_2PHAL$ (4 mol %), $K_2OsO_2(OH)_4$ (0.6 mol %) and $NaHCO_3$ (32.4 mmol) in a mixture of *t*-BuOH/water (110 mL, 1:1 v/v) according to the procedure and work-up described previously to afford diol **28a** (1.56 g ca. 80%, 83% ee by $^{19}F\{^1H\}$ NMR, L-(+)-DIPT/$CDCl_3$).

1 mol % osmium/5 mol % $(DHQ)_2AQN$ ligand

Crude diol **28a** was prepared from a solution of fluoroester **25** (1.5 g, 10.2 mmol), $K_3Fe(CN)_6$ (10 g, 30.6 mmol), K_2CO_3 (4.2 g, 30.6 mmol), $MeSO_2NH_2$ (975 mg, 10.2 mmol), $(DHQ)_2AQN$, (5 mol %, 435 mg), $K_2OsO_4{\cdot}2H_2O$ (2 mol %, 37 mg) in a mixture of *t*-BuOH and water (70 mL, 1:1 v/v) according to the procedure and work-up

described previously to afford diol **28a** (640 mg, 59%, 95% ee by $^{19}F\{^{1}H\}$ NMR, L-(+)-DIPT/$CDCl_3$).

Preparation of *n*-propyl (2*S,3*S**)-dibenzyloxy-4-fluorobutanoate ((±)-29c)**

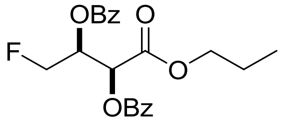

Benzoic anhydride (1.13 g, 5 mmol), DMAP (49 mg, 0.4 mmol) and poly(vinylpyridine) (2.5 g, 2.5 mmol) were added to a solution of diol **28c** (360 mg, 2 mmol) in DCM (20 mL) at room temperature. The mixture was then shaken at room temperature overnight. Analysis of the crude mixture by ^{19}F NMR showed the reaction had not gone to completion. Benzoic anhydride (0.57 g, 2.5 mmol) was added and the mixture was shaken at room temperature for a further 4 hours. Analysis of an aliquot by ^{19}F NMR confirmed full conversion of the starting material had taken place. The mixture was diluted with diethyl ether (10 mL), filtered, and the precipitate washed with Et_2O (10 mL). The filtrate was then washed with $NaHCO_3$ (3 × 15 mL of a saturated aqueous solution), brine (15 mL), dried ($MgSO_4$) and concentrated in vacuo to afford a brown oil (700 mg, ca. 90% purity). Purification by Flash chromatography (Buchi Sepacore, 10% diethyl ether in hexane) afforded dibenzoate **29c** as a colourless syrup (622 mg, 80%): R_f (10% diethyl ether in hexane) 0.29; δ_H (400 MHz, $CDCl_3$) 8.11 (m incl. app. d, J = 17.7, 2H), 8.09 (m incl. app. d, J = 17.7, 2H), 7.64-7.56 (m, 2H), 7.50-7.42 (m, 4H), 5.96-5.86 (dq, J = 13.7, J = 3.7, 1H), 5.70 (d, J = 3.7, 1H), 4.78 (dqd, $^{2}J_{H\text{-}F}$ = 46.5, J = 9.7, J = 5.6, 2H) , 4.14 (t, J = 6.7, 2H), 1.66-1.56 (m, 2H), 0.87 (t, J = 7.8, 3H); δ_C (100 MHz, $CDCl_3$) 166.8, 165.4, 165.2, 133.7, 133.6, 130.0, 129.9, 128.6, 128.5, 80.2 (d, $^{1}J_{C\text{-}F}$ = 174.2), 70.6 (d, $^{3}J_{C\text{-}F}$ = 4.4), 70.3 (d, $^{2}J_{C\text{-}F}$ = 23.4), 67.7, 21.8, 10.2; δ_F (376 MHz, $CDCl_3$) -230.3

(td, $^2J_{F-H}$ = 47.0, $^3J_{F-H}$ = 12.6); ν_{max}(film)/cm^{-1} 3070, 2970, 2674, 1971, 1915, 1729, 1602, 1452, 1289, 1104, 1026, 935, 804, 712; HRMS (ES$^+$, [M + NH$_4$]$^+$) calcd. for $C_{21}H_{21}FO_6NH_4$ 406.1660, found 406.1663, (ES$^+$, [2M + NH$_4$]$^+$) calcd. for $C_{42}H_{42}F_2O_{12}NH_4$ 794.2983, found 794.2990.

Preparation of *n*-propyl (2*S*,3*S*)-dibenzyloxy-4-fluorobutanoate (29b)

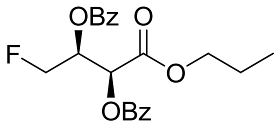

Was prepared from benzoic anhydride (127 mg, 0.56 mmol), DMAP (7 mg, 0.056 mmol) poly(vinylpyridine) (280 mg, 0.28 mmol), and diol **28b** (50 mg, 0.28 mmol) in DCM (3 mL) according to the procedure, work-up and purification described previously to afford dibenzoate **29b** as a colourless oil (88 mg, 81%). Data were identical to those for **29c** apart from; [α]$_D$ +47.6 (*c* 0.55, $CHCl_3$); HPLC (Chiralcel OD-H column, hexane/iPrOH 98/2, 1 mL min^{-1}, 280 nm): t_R (major) 18.1 min, 91% ee.

Preparation of *n*-propyl (2*R*,3*R*)-dibenzyloxy-4-fluorobutanoate (29a)

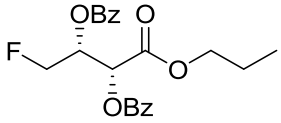

Was prepared from benzoic anhydride (127 mg, 0.56 mmol), DMAP (7 mg, 0.056 mmol), poly(vinylpyridine) (280 mg, 0.28 mmol), diol **28a** (50 mg, 0.28 mmol) in DCM (3 mL) according to the procedure, work-up and purification described previously to afford dibenzoate **29a** as a colourless oil (86 mg, 79%). Data were identical to those for **29c** apart from; [α]$_D$ -50.4 (*c* 0.5, $CHCl_3$); HPLC (Chiralcel OD-H column, hexane/iPrOH 98/2, 1 mL min^{-1}, 280 nm): t_R (major) 11.7 min, 83% ee.

Preparation of (4*S,5*S**)-5-(fluoromethyl)-[1,3,2]-dioxathiolane-4-(*n*-propyloxycarbonyl)-2,2-dioxide ((±)-32c); direct sequence from 25**

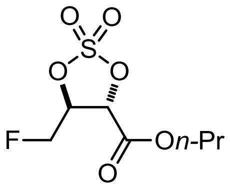

A solution of NMO (384 mg, 3.3 mmol) in water (0.5 mL) was added to a 1:1 mixture of *t*-BuOH/acetone (4 mL) at 0 °C. Ester **25** (239 mg, 1.64 mmol) was then added and the mixture was allowed to stir for 15 minutes, and then OsO_4 (330 µL of a 2.5 wt % solution in *t*-BuOH) was added dropwise by syringe over 20 minutes. The mixture was allowed to continue stirring and slowly warmed to room temperature overnight. The progress of this reaction was monitored by the ^{19}F NMR spectra. The reaction was quenched by the addition of sodium sulfite (1.7 g), stirred for 30 minutes and eluted through a pad of Celite with EtOAc/MeOH (30 mL a 1:1 mixture). The filtrate was then concentrated in vacuo to afford the diol **28c** (δ_F $^{19}F\{^1H\}$ -223.3).
Crude diol **28c** was taken up in dry DCM (6 mL), cooled to 0 °C and treated with pyridine (370 µL, 4.6 mmol) and $SOCl_2$ (170 µL, 3.3 mmol). After stirring for 15 minutes, the reaction was quenched with water (10 mL) and extracted with DCM (3 × 10 mL). The combined organic extracts were then washed with brine (10 mL), dried ($MgSO_4$) and concentrated in vacuo to afford crude (±)**-31c** as a mixture of diastereoisomers (δ_F $^{19}F\{^1H\}$ -225.8 and -234.0).
Crude cyclic sulfite (±)**-31c** was taken up in a mixture of acetonitile (8 mL) and water (1.5 mL), cooled to 0 °C and solid $NaIO_4$ (491 mg, 2.3 mmol) followed by $RuCl_3$ (catalytic, ~ 1 drop from a capillary) were added. After stirring for 10 minutes, the reaction mixture was diluted with Et_2O (10 mL) and filtered through a pad of Celite. The organic filtrate was then washed with water (10 mL), $NaHCO_3$ (10 mL of a

saturated aqueous solution), brine (10 mL), dried ($MgSO_4$) and concentrated in vacuo to afford cyclic sulfate (±)-**32c** (δ_F $^{19}F\{^1H\}$ -223.4). Purification by flash chromatography (Buchi Sepacore, 30% ethyl acetate in hexane) afforded (±)-**32c** as a colourless oil (117 mg, 29% from **25**): R_f (30% ethyl acetate in hexane) 0.42; δ_H (400 MHz, $CDCl_3$) 5.21 (d, J = 7.3, 1H), 5.14 (dddd $^3J_{H\text{-}F}$ = 21.7, J = 7.3, = 3.3, 2.2, 1H), 4.9–4.66 (m, 2H) 4.25-4.21 (m, 2H), 1.78-1.73 (m, 2H), 0.98 (t, J = 7.4, 3H); δ_C (100 MHz, $CDCl_3$) 164.7, 79.2 (d, $^1J_{C\text{-}F}$ = 181.3), 74.7 (d, $^3J_{C\text{-}F}$ = 6.8), 80.5 (d, $^2J_{C\text{-}F}$ = 19.9), 69.1, 21.7, 10.1; δ_F (376 MHz, $CDCl_3$) -223.4 (ddd, $^2J_{F\text{-}H}$ = 47.3, $^2J_{F\text{-}H}$ = 45.9, $^3J_{F\text{-}H}$ = 21.7); ν_{max}(film)/cm^{-1} 3670, 3527, 2975, 2885, 1771, 1745; HRMS (EI, $[M + H]^+$) calcd. for $C_7H_{12}FO_6S$ 243.0333, found 243.0341; Anal. Calcd. for $C_7H_{11}FO_6S$: C, 34.71; H, 4.58; S, 13.24. Found: C, 35.05; H, 4.69; S, 13.25.

Preparation of (4*S*,5*S*)-5-(fluoromethyl)-[1,3,2]-dioxathiolane-4-(*n*-propyloxycarbonyl)-2,2-dioxide ((+)-32b) from 28b

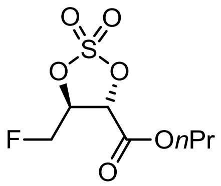

Was prepared from diol **28b** (792 mg, 4.4 mmol), dry DCM (20 mL), pyridine (1.36 µL, 17.6 mmol), $SOCl_2$ (615 µL, 8.8 mmol) then $NaIO_4$ (1.3 g, 6.2 mmol), $RuCl_3$ (catalytic ~2–3 drops from a capillary), acetonitrile (25 mL) and water (5 mL) according to the procedure, work-up and purification described previously to afford cyclic sulfate (+)-**32b** (340 mg, 29% yield). Data were identical to those described previously for (±)-**32c** apart from; $[\alpha]_D$ +23.4 (c 1.0, $CDCl_3$); HPLC (Chiralcel OD-H column, hexane/iPrOH 85/15, 1 mL min^{-1}, 280 nm): t_R (major) 14.5 min, 92% ee.

Preparation of (4*R*,5*R*)-5-(fluoromethyl)-[1,3,2]-dioxathiolane-4-(*n*-propyloxycarbonyl)-2,2-dioxide ((−)-32a) from 28a

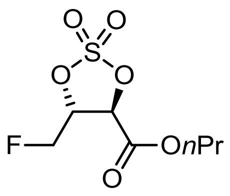

Was prepared from diol **28a** (792 mg, 4.4 mmol), dry DCM (20 mL), pyridine (1.36 μL, 17.6 mmol), $SOCl_2$ (615 μL, 8.8 mmol) then $NaIO_4$ (1.3 g, 6.2 mmol), $RuCl_3$ (catalytic ~2–3 drops from a capillary), acetonitrile (25 mL) and water (5 mL) according to the procedure, work-up and purification described previously to afford cyclic sulfate **(−)-32a** (553 mg, 38% yield). Data were identical to those described previously for **(±)-32c** apart from; $[\alpha]_D$ -20.5 (*c* 1.0, $CDCl_3$); HPLC (Chiralcel OD-H column, hexane/iPrOH 85/15, 1 mL min^{-1}, 280 nm): t_R (major) 13.2 min, 80% ee.

Preparation of *n*-propyl (2*R*)-benzyloxy-4-fluoro-(3*S*)-hydroxybutanoate (33b)

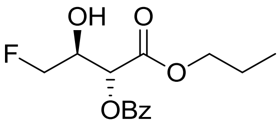

Crude cyclic sulfate (+)-**32b** was prepared from enriched diol **28b** (90 mg, 0.5 mmol), dry DCM (1.5 mL), pyridine (180 μL, 2.3 mmol), $SOCl_2$ (75 μL, 1 mmol), $NaIO_4$ (149 mg, 0.75 mmol), $RuCl_3$ (catalytic ~1 drop from a capillary), acetonitrile (3 mL) and water (1 mL) and according to the procedure and work-up described previously. Ammonium benzoate (119 mg, 1 mmol) was added to a solution of (+)-**32b** in acetone (2 mL) and the mixture was allowed to stir at room temperature overnight. After confirmation that the reaction was complete by ^{19}F NMR, the reaction was concentrated in vacuo and taken up in Et_2O (2.5 mL). A solution of sulfuric acid (2.5 mL of a 20% v/v aqueous solution) was then added dropwise and the resulting

mixture was allowed to stir at room temperature overnight. After the consumption of the starting material had been confirmed by ^{19}F NMR, the mixture was partitioned between ether (10 mL) and water (10 mL). The aqueous layer was separated, neutralised with solid $NaHCO_3$ and extracted with ether (2 × 5 mL). The combined organic extracts were then washed with brine (10 mL), dried ($MgSO_4$) and concentrated under reduced pressure to yield the crude material as an off white semi-solid (222 mg). Purification by Flash chromatography (Buchi Sepacore, 10% diethyl ether in hexane then 0–8% methanol in dichloromethane) afforded monobenzoate **33b** as a colourless oil (85 mg, 60% yield): R_f (10% methanol in dichloromethane) 0.39; $[\alpha]_D$ +2.9 (*c* 1.25, $CDCl_3$); δ_H (400 MHz, $CDCl_3$) 8.12-8.06 (m, 2H), 7.64-7.59 (m, 1H), 7.50-7.45 (m, 2H), 5.40 (d, J = 5.6, 1H), 4.64 (ddd, $^2J_{H-F}$ = 47.0, J = 4.5, J = 1.5, 1H), 4.63 (ddd, $^2J_{H-F}$ = 47.0, J = 5.0, J = 2.0, 1H), 4.50-4.42 (dq, J = 18.7, J = 4.8, 1H), 4.19 (t, J = 6.6, 2H), 1.73-1.64 (m incl. J = 6.6, J = 7.6, 2H), 0.93 (t, J = 7.6, 3H); δ_C (100 MHz, $CDCl_3$) 168.1, 165.5, 133.7, 130.2, 129.9, 128.8, 128.6, 128.4, 82.9 ($^1J_{C-F}$ = 171.3), 72.6 ($^3J_{C-F}$ = 5.9), 70.1 ($^2J_{C-F}$ = 20.5), 67.6, 21.8, 10.2; δ_F (376 MHz, $CDCl_3$) -232.0 (td, $^2J_{F-H}$ = 47.0, $^3J_{F-H}$ = 18.4); ν_{max}(film)/cm^{-1} 3482 (br), 2964, 1973, 1725, 1604, 1451, 1263, 1107, 709; HRMS (ES^+, $[M + H]^+$) calcd. for $C_{14}H_{17}FO_5NH_4$ 302.1398, found 302.1403, (ES^+, $[2M + NH_4]^+$) calcd. for $C_{28}H_{34}F_2O_{10}NH_4$ 591.2012, found 591.2013.

Preparation of n-propyl (2*R*,3*S*)-dibenzoyloxy-4-fluorobutanoate (34b); direct sequence from 28b

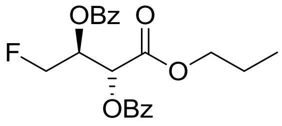

Cyclic sulfate (+)-**32b** was prepared from diol **28b** (245 mg, 1.36 mmol), dry DCM (4 mL), pyridine (487 μL, 6.1 mmol), $SOCl_2$ (204 μL, 2.7 mmol) then $NaIO_4$ (435 mg, 2.8 mmol), $RuCl_3$ (catalytic couple of drops from a capillary) acetonitrile (9 mL) and water (3 mL) according to the procedure and work-up procedures described previously. The crude product was taken up in acetone (2 mL) and ammonium benzoate (323 mg, 2.7 mmol) was added. The mixture was allowed to stir at room temperature overnight. After confirmation that the reaction was complete by ^{19}F NMR, the reaction was concentrated in vacuo and taken up in Et_2O (2.5 mL). A solution of sulfuric acid (2.5 mL, of a 20% v/v aqueous solution) was then added dropwise and the resulting mixture allowed to stir at room temperature overnight. After consumption of the starting material had been confirmed by ^{19}F NMR, the mixture was diluted with ether (10 mL) and water (10 mL). The aqueous layer was separated, neutralized with solid $NaHCO_3$ and extracted with ether (2 × 5 mL). The combined organic extracts were then washed with brine (10 mL), dried ($MgSO_4$) and concentrated under reduced pressure to yield the crude monobenzoate **33b**.

Product **33b** was taken up in DCM (14 mL), treated with benzoic anhydride (370 mg, 1.63 mmol), DMAP (33 mg, 20 mol %) and PVP (1.4 g) and shaken overnight at room temperature. Analysis of an aliquot by ^{19}F NMR confirmed that the reaction had gone to completion. The mixture was diluted with Et_2O (25 mL), filtered, and the precipitate washed with Et_2O (20 mL). The filtrate and washings were combined and washed with $NaHCO_3$ (3 × 20 mL of a saturated aqueous solution), brine (20 mL),

dried ($MgSO_4$) and concentrated in vacuo. Purification by flash chromatography (Buchi Sepacore, 10% diethyl ether in hexane) afforded dibenzoate **34b** as a colourless oil (170 mg, 32% from **28b** over 3 steps). R_f (10% diethyl ether in pet. ether 40–60 °C) 0.21; $[\alpha]_D$ -4.5 (*c* 1.1, $CDCl_3$), δ_H (400 MHz, $CDCl_3$) 8.10-8.03 (m, 4H), 7.64-7.52 (m, 2H), 7.50-7.43 (m, 4H), 5.92-5.88 (m, 1H), 5.69 (dd, *J* = 4.6, *J* = 1.1, 1H), 4.94-4.80 (m incl. app. d, $^2J_{H\text{-}F}$ = 46.7, 1H), 4.24-4.14 (m, 2H), 1.71-1.62 (m, 2H), 0.92 (t, *J* = 7.3, 3H); δ_C (100 MHz, $CDCl_3$) 167.9, 165.3, 165.25, 133.7, 133.6, 130.0, 129.9, 129.1, 128.8, 128.6, 128.4, 80.5 ($^1J_{C\text{-}F}$ = 175.6), 70.8 ($^3J_{C\text{-}F}$ = 5.8), 70.9 ($^2J_{C\text{-}F}$ = 22.0), 67.7, 21.8, 10.2; δ_F (376 MHz, $CDCl_3$) -231.0 (td, $^2J_{F\text{-}H}$ = 46.7, $^3J_{F\text{-}H}$ = 16.7); ν_{max}(film)/cm^{-1} 3056, 2948, 1733, 1602, 1454, 1263, 1099, 712; HRMS (ES^+, $[M + NH_4]^+$) calcd. for $C_{21}H_{21}FO_6NH_4$ 406.1667, found 406.1660; HPLC (Chiralcel OD-H column, hexane/*i*PrOH 98/2, 1mL min^{-1}, 280 nm): t_R (major) 11.2 min, 95% ee.

Preparation of n-propyl (2*S*,3*R*)-dibenzoyloxy-4-fluorobutanoate (34a)

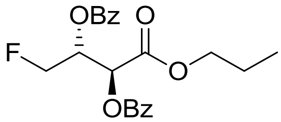

Dibenzoate **34a** was prepared from diol **28a** (86 mg, 0.48 mmol), dry DCM (1.4 mL), pyridine (175 μL, 2.2 mmol), $SOCl_2$ (75 μL, 1 mmol) then $NaIO_4$ (155 mg, 1 mmol) catalytic $RuCl_3$ (couple of drops from a capillary ~5–10 mg), acetonitrile (3 mL), water (1 mL) then ammonium benzoate (119 mg, 1 mmol), benzoic anhydride (136 mg, 0.6 mmol), DMAP (12 mg, 20 mol %), PVP (0.5 g) and DCM (5 mL) according to the procedure described previously to afford dibenzoate **34a** (65 mg, 34% over 3 steps). Data were identical to those described previously for **34b** apart from; $[\alpha]_D$ +4.4 (*c* 1.3, $CDCl_3$); HPLC (Chiralcel OD-H column, hexane/iPrOH 98/2, 1 mL min^{-1}, 280 nm): t_R (major) 12.7 min, 78% ee.

Preparation of *n*-propyl (2*R*,3*S*)-dihydroxy-4-fluorobutanoate (35b)

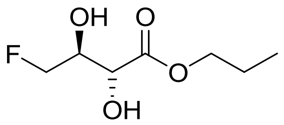

Cyclic sulfate **32b** was prepared from diol **28b** (432 mg, 2.4 mmol), dry DCM (7.5 mL), pyridine (895 μL, 11.1 mmol), $SOCl_2$ (357 μL, 4.8 mmol), $NaIO_4$ (770 mg, 3.6 mmol) and catalytic $RuCl_3$ (couple of drops from a capillary ~5–15 mg) according to the procedure and work-up procedures described previously. Crude **32b** was taken up in DMF (10 mL) and sodium nitrite (828 mg, 12 mmol) was added in one portion at room temperature, and the mixture was allowed to stir overnight. After confirmation of consumption of the starting materials by ^{19}F NMR, the reaction was diluted with water (10 mL) and extracted with ethyl acetate (3 × 10 mL). The combined organic extracts were then dried (Na_2SO_4) and concentrated under reduced pressure. The resulting yellow oil was then taken up in diethyl ether (4 mL) and sulfuric acid added dropwise (4 mL of a 20% v/v aqueous solution). The reaction mixture was diluted with diethyl ether (10 mL) and washed with $NaHCO_3$ (10 mL of a saturated solution) then brine (10 mL). The organic layer was then isolated, dried ($MgSO_4$) and concentrated under reduced pressure. Purification by flash chromatography (Buchi Sepacore, 0–50% diethyl ether in pet. ether 40–60 °C) afforded **35b** as a colourless oil (52 mg, 12% from **28b**): R_f (100% diethyl ether in pet. ether 40–60 °C) 0.49; δ_H (400 MHz, $CDCl_3$) 4.64-4.45 (m incl. app. dd, J = 47.0, J = 5.1, 2H), 4.32 (d, J = 4.0, 1H), 4.22-4.18 (m incl. app. t, J = 6.6, 2H), 4.18-4.12 (m, 1H), 3.32 (br s, 1H), 2.85 (br s, 1H), 1.76-1.67 (m, 2H), 0.97 (t, J = 7.3, 3H); δ_C (100 MHz, $CDCl_3$) 172.2, 82.7 (d, $^1J_{C\text{-}F}$ = 169.8), 71.7 (d, $^2J_{C\text{-}F}$ = 20.5), 69.9 (d, $^3J_{C\text{-}F}$ = 5.9), 68.0, 21.8, 10.2; δ_F (376 MHz, $CDCl_3$) -230.2 (td, $^2J_{F\text{-}H}$ = 47.0, $^3J_{F\text{-}H}$ = 16.1);

ν_{max}(film)/cm^{-1} 3478, 2974, 2556, 1748, 1652, 1296, 1057, 850; HRMS (FAB, $[M+NH_4]^+$) calcd. for $C_7H_{17}O_4NF$ 198.1136, found 198.1134; $^{19}F\{^1H\}$ (376 MHz, 1:1 di*iso*propyl L-tartrate:$CDCl_3$) 94% ee; $[\alpha]_D$ +33.4 (*c* 1.04, $CDCl_3$).

Preparation of (2*S*,3*R*)-propyl 4-fluoro-2,3-dihydroxybutanoate (35a)

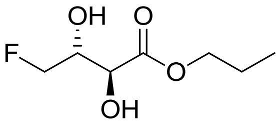

Diol **35a** was prepared from diol **28a** (432 mg, 2.4 mmol), dry DCM (7.5 mL), pyridine (895 μL, 11.1 mmol), $SOCl_2$ (357 μL, 4.8 mmol), $NaIO_4$ (770 mg, 3.6 mmol), catalytic $RuCl_3$ (couple of drops from a capillary ~5–15mg) then sodium nitrite (828 mg, 12 mmol) and DMF (10 mL) and according to the procedure described previously to afford *anti*-diol **35a** as a colourless oil (41 mg, 9% from **28a**). The data were identical to those obtained for **35b** apart from; δ_F ^{19}F $\{^1H\}$ (376 MHz, 1:1 di*iso*propyl L-tartrate:$CDCl_3$) 95% ee; $[\alpha]_D$ -34.0 (*c* 0.99, $CDCl_3$).

Preparation of *n*-propyl (2*R*,3*R*)-*O*-cyclohexylidene-4-fluorobutanoate (36a)

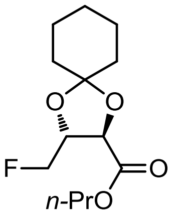

Diol **28a** (314 mg, 1.7 mmol) was taken up in a mixture of cyclohexanone (200 mg, 2.0 mmol) and trimethyl orthoformate (216 mg, 2.0 mmol) in ethyl acetate (5 mL) and stirred for 15 minutes at room temperature. The yellow solution was then treated with boron trifluoride diethyl etherate (260 μL, 2.04 mmol). A colour change to dark orange was observed and the mixture was stirred at room temperature for 48 hours.

The mixture was then diluted with ethyl acetate (30 mL) and the organic phase was washed with $NaHCO_3$ (30 mL of a saturated aqueous solution), brine (30 mL), dried ($MgSO_4$) and concentrated in vacuo to afford an orange oil. Purification by flash chromatography (Buchi Sepacore, 10% diethyl ether in hexane) afforded **36a** as a colourless oil (96 mg, 21%): R_f (10% diethyl ether in hexane) 0.37; $[\alpha]_D$ +26.8 (c 1.2, $CDCl_3$); δ_H (400 MHz, $CDCl_3$) 4.76-4.32 (m, 4H), 4.21-4.10 (m, 2H), 1.75-1.55 (br m, 10H), 1.45-1.37 (br m, 2H), 0.96 (t, J = 7.1, 3H); δ_C (100 MHz, $CDCl_3$) 170.7, 112.8, 82.1 (d, $^1J_{C\text{-}F}$ = 175.6), 77.3 (d, $^2J_{C\text{-}F}$ = 20.5) 74.1 (d, $^3J_{C\text{-}F}$ = 7.3), 67.2, 36.2, 35.0, 25.0, 23.8, 23.6, 21.9, 10.3; δ_F (376 MHz, $CDCl_3$) -230.45 (td, $^2J_{F\text{-}H}$ = 47.0, J = 21.2); ν_{max}(film)/cm^{-1} 2944, 1760, 1728, 1449, 1362, 1288, 1240, 1159, 1066, 1021, 908, 842, 829, 792, 652; *m/z* (EI^+) 261 (7), 231 (43), 217 (100), 175 (24); HRMS (ES^+, $[M + H]^+$) calcd. for $C_{13}H_{22}FO_4$ 261.1497, found 261.1497.

n-Propyl (2*S*,3*S*)-*O*-cyclohexylidene-4-fluorobutanoate (36b)

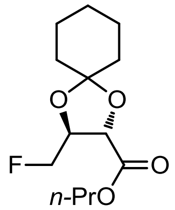

Was prepared from diol **28b** (1.09 g, 6.1 mmol), cyclohexanone (598 mg, 6.1 mmol), boron trifluoride diethyl etherate (927 µL, 7.3 mmol) and ethyl acetate (5 mL) and according to the procedure, work-up and purification described previously to afford acetal **36b** was afforded (393 mg, 19%). The data were identical to those obtained for **36a** apart from; $[\alpha]_D$ -30.0 (*c* 0.8, $CDCl_3$).

Preparation of (2*R*,3*R*)-*O*-cyclohexylidene-4-fluorobutan-1-ol (37a)

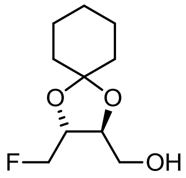

A solution of acetal **36a** (1.02 g, 3.9 mmol) in dry DCM (40 mL) was cooled to −78 °C under an atmosphere of nitrogen. DIBAL-H (9.75 mmol, 8.9 mL of a 1.1 M solution in cyclohexane) was then added dropwise over 20 minutes and the reaction was allowed to warm slowly to room temperature overnight under a nitrogen atmosphere. The mixture was diluted with EtOAc (60 mL) and brine (60 mL) forming a viscous emulsion which was filtered through a pad of wet Celite. The organic layer in the filtrate was isolated and the aqueous layer was extracted with EtOAc (2 × 50 mL). The combined organic extracts and the original layer were then dried ($MgSO_4$) and concentrated in vacuo to afford a yellow oil. Purification by flash chromatography (Buchi Sepacore, 0–40% ethyl acetate in hexane) afforded alcohol **37a** as a pale yellow oil (300 mg, 38% yield): R_f (50% ethyl acetate in hexane) 0.65; $[\alpha]_D$ -3.2 (*c* 0.9, $CDCl_3$), δ_H (400 MHz, $CDCl_3$) 4.65-4.43 (ddd, $^2J_{H\text{-}F}$ = 47.5, *J* = 4.1, *J* = 3.5, 2H), 4.20-4.10 (m, 1H), 4.08-4.04 (m, 1H), 3.90-3.83 (m incl. app. d, *J* = 12.1, 1H), 3.70-3.63 (m, 1H), 1.96-1.89 (m, 1H), 1.69-1.56 (envelope, 8H), 1.45-1.37 (m, 2H); δ_C (100 MHz, $CDCl_3$) 110.5, 82.4 (d, $^1J_{C\text{-}F}$ = 172.7), 77.8 (d, $^3J_{C\text{-}F}$ = 5.9), 75.2 (d, $^2J_{C\text{-}F}$ = 20.5), 61.9, 36.7, 36.3, 25.0, 23.85, 23.75; δ_F (376 MHz, $CDCl_3$) -230.8 (td, $^2J_{F\text{-}H}$ = 47.0, $^3J_{F\text{-}H}$ = 20.7); ν_{max}(film)/cm^{-1} 3446br, 2938, 2863, 1450, 1366, 1282, 1117, 940, 845; *m/z* (EI^+) 205 (84), 175 (44), 161 (100), 99 (18), 81 (22), 69 (24), 55 (56); HRMS (ES^+, $[M + H]^+$) calcd. for $C_{10}H_{18}FO_3$ 205.1234, found 205.1233.

Preparation of (2*S*,3*S*)-*O*-cyclohexylidene-4-fluorobutan-1-ol (37b); direct preparation from 25

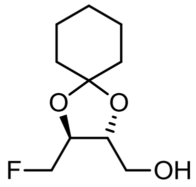

Crude diol **28b** was prepared from a solution of fluoroester **25** (2.19 g, 15 mmol), AD-mix β (21 g), methyl sulfonamide (1.425 g, 15 mmol), $(DHQD)_2PHAL$ (4 mol %), $K_2OsO_2(OH)_4$ (0.6 mol %) and $NaHCO_3$ (45 mmol) in 1:1 *t*-BuOH/water (150 mL) according to the procedure and work-up described previously to afford crude diol **28b** (2.43 g, ~89%).

Acetal **36b** was prepared from crude diol **28b** (2.43 g, 13.5 mmol), cyclohexanone (1.6 g, 16.2 mmol), boron trifluoride diethyl etherate (2.3 mL, 16.2 mmol) and ethyl acetate (10 mL) according to the procedure described previously, to afford crude acetal **36b** was afforded (2.1 g, ~59%).

A solution of the crude ester **36b** (2.1 g, 8.1 mmol) in dry DCM (140 mL) was cooled to −78 °C under an atmosphere of nitrogen. DIBAL-H (20.3 mmol, 18.4 mL of a 1.1 M solution in cyclohexane) was then added dropwise over 20 minutes and the reaction was allowed to warm slowly to room temperature overnight under a nitrogen atmosphere. The mixture was diluted with EtOAc (100 mL) and brine (100 mL) forming a viscous emulsion which was filtered through a pad of wet Celite. The organic layer in the filtrate was isolated and the aqueous layer was extracted with EtOAc (2 × 100 mL). The combined organic extracts and the original layer were then dried ($MgSO_4$) and concentrated in vacuo to afford a yellow oil. Purification by flash chromatography (Buchi Sepacore, 0–40% ethyl acetate in hexane) afforded alcohol

37b as a pale yellow oil (758 mg, 25% over 3 steps); The data were identical to those obtained for **37a** apart from; $[\alpha]_D$ +2.9 (*c* 0.9, $CDCl_3$).

Preparation of ethyl (4*R*,5*R*)-*O*-cyclohexylidene-6-fluoro-hex-2-enoate (39a)

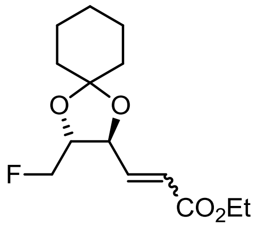

Alcohol **37a** and (carbethoxymethylene)triphenylphosphorane (780 mg, 2.24 mmol) were taken up in a mixture of DCM (4.2 mL) and DMSO (0.7 mL) at room temperature and allowed to stir at room temperature for 5 minutes. Solid Dess–Martin periodinane (93 mg, 1.23 mmol) was added causing an instant colour change to yellow. The reaction was stirred for 30 minutes then diluted with Et_2O (50 mL) and the organic phase washed with $NaHCO_3$ (3 × 50 mL of a saturated aqueous solution), dried ($MgSO_4$) and concentrated in vacuo to a viscous orange gum. Purification by flash chromatography (Buchi Sepacore, 0–50% ethyl acetate in hexane) afforded alkenoate **39a** as a 4:1 mixture of *E* and *Z* diastereoisomers (114 mg, 74%). Re-purification by flash chromatography (Buchi Sepacore, 0-50% ethyl acetate in hexane) afforded *E*-**39a** as a clear oil (57 mg, 37%): R_f (50% diethyl ether in hexane) 0.56; $[\alpha]_D$ +6.5 (*c* 1.2, $CDCl_3$), δ_H (400 MHz, $CDCl_3$) 6.91 (dd, *J* = 15.7, *J* = 5.6, 1H), 6.17 (dd, *J* =15.7, *J* = 1.5, 1H), 4.68-4.43 (m, 3H), 4.22 (q, *J* = 7.3, 2H), 4.01-3.97 (m, 1H), 1.70-1.58 (envelope, 8H), 1.57-1.45 (m, 2H), 1.30 (t, *J* = 7.1, 3H); δ_C (100 MHz, $CDCl_3$) 165.8, 143.4, 123.2, 111.4, 81.3 (d, $^1J_{C-F}$ = 174.2), 78.7 (d, $^2J_{C-F}$ = 20.5), 75.6 (d, $^3J_{C-F}$ = 7.3), 60.7, 36.3, 36.2, 25.0, 23.8, 23.75, 14.2; δ_F (376 MHz, $CDCl_3$) -231.0 (td, $^2J_{F-H}$ = 47.0, $^3J_{F-H}$ = 20.7); ν_{max}(film)/cm^{-1} 3425, 2939, 2863, 1723, 1664, 1450, 1368, 1303, 1278, 1178, 1130, 1034, 976, 908, 847; *m/z* (EI^+) 273 (26),

243 (26), 229 (56), 199 (10), 175 (11), 129 (20), 81 (22); HRMS (EI^+, $[M]^+$) calcd. for $C_{14}H_{21}FO_4$ 272.1418, found 272.1419; 82% ee by $^{19}F\{^1H\}$ NMR, L-(+)-DIPT/$CDCl_3$, followed by a mixed fraction (*E*- and *Z*-diastereoismers, 57 mg, 37%).

References

S1. Kumar, B.; Verma, R. K., *Synth. Commun.,* **1984**, *14*, 1359.

S2. Fedorynski, M.; Ziolkowska, W.; Jonczyk, A., *J. Org. Chem.,* **1993**, *58*, 6120.
